# The Impact of Unplanned School Closures on Adolescent Behavioral Health During the Covid-19 Pandemic in Malaysia

**DOI:** 10.3389/fpubh.2021.639041

**Published:** 2021-06-07

**Authors:** Muhammad Syawal Amran, Khairul Azhar Jamaludin

**Affiliations:** Faculty of Education, National University of Malaysia, Bangi, Malaysia

**Keywords:** unplanned school closure, behavioral health, adolescent, health education, COVID-19

## Abstract

School closures were implemented as a public health intervention to reduce the risk of infection from COVID-19. However, prolonged school closure is likely to impact adolescents' behavioral health due to the extreme change in routine. The current study aimed to explore adolescents' behavioral health experiences during the beginning of the outbreak of the COVID-19 pandemic. This study was done using qualitative methods to interview 15 adolescent participants (*n* =15) from low-income households in Malaysia. The study lasted for 2 months amidst the outbreak and data were collected via online based on focus group discussions. The results revealed that adolescents faced four main themes of experience during the COVID-19 pandemic: Alteration of sleep patterns, stress-related fatigue, dysfunctional eating patterns and lack of physical activity. This first-hand experience shows that knowledge and skills of adolescents' behavioral practices during outbreak deserves attention. This research stresses the role of family, schools, and media in addressing the health communication gap among adolescents to help them adapt in these new norms.

## Introduction

The coronavirus (COVID-19) pandemic has spread globally and was announced as a Public Health Emergency International Concern (PHEIC). This crisis has caused numerous countries, including Malaysia, to restrict movement by means of government-enforced Movement Control Order (MCO). MCO's had vast effects on various sectors such as economy, tourism and trade, both domestically and in terms of global trade. The efforts to prevent the spread of COVID-19 also pushed many countries around the world to take proactive measures to temporarily close educational institutions. A report from the United Nation's Educational, Scientific and Cultural Organization (1) estimated that more than 60% of the world's children and adolescents were affected by these school closures.

School is one of the defining pillars of the adolescent experience, shaping how adolescents access education, socialize with peers, access behavioral health services, and exercise in recreational sport and activities. Naturally, MCOs and school closure significantly interrupt typical adolescent lifestyles. What is worrying is that the face-to-face interactions with those outside the home become almost impossible, as everyone is restricted in movement and required to stay at home and physically isolate themselves for a certain period of time. According to Bulut (2), face-to-face interaction has a positive effect on students' well-being. An additional difficulty is that adolescence is a unique stage of experimentation and exploration. Adolescents prefer to engage with the world freely, often electing to spend more time with their peers than with their family. This interruption may lead to other challenges impacting well-being (3).

Another issue which contributes to worsening stress levels and mental well-being is the problem of limited house space. With quarantine and movement control orders disruption reaching into every facet of daily life, from employment to education to grocery shopping to socializing, multiple family members are having to live together in small living spaces, as they are unable to leave freely (4). For students from low social economic status, this situation will leave a bigger impact on how they live their lives on daily basis. Having a smaller space to be shared with other family members will leave them to feel discomfort and lead to depression. A recent study by Amerio et al. (5) indicated that students with moderate–severe and severe depressive symptoms live in small houses/apartments.

Adolescents feel upset due to the disruption of various daily activities, disruption in the original plan for learning and concerns about the uncertain future (6, 7). Their failure to adapt to the daily routine and maximize their time and space for learning and activities affects their behavioral well-being. In addition, their confinement to the home environment also interferes with their daily routine. It eventually creates a sense of boredom and also relates to excessive calorie intake and irregular sleeping pattern.

Meanwhile, prolonged exposure such as reading and listening to COVID-19 updates from mass media and social media also may lead to pressure. These circumstances lead adolescents to engage in unhealthy lifestyle choices, such as overeating and so-called “emotional eating,” as well as excessive sleeping which may raise the risk for obesity among adolescents (8, 9). Prolonged isolation at home could impact many areas of adolescent health, including behavioral, mental and physical health (10). The purpose of this research isto explore the impact of unplanned school closure on adolescents' behavioral health during COVID-19 pandemic outbreak in Malaysia

## Methodology

A qualitative focus group design was selected to capture behavioral health experiences in adolescents who stayed at home during Movement Control Order (MCO) due to school closure in Malaysia. This research was conducted in it's entirely during the first months of the states of alarm in Malaysia (which began on 18 March 2020). The state of alarm implied the isolation and confinement of the entire population, the closing of all schools and educational institutions as well as closing non-essential business. The people were only allowed to out to the street for essential matters such as shopping for essential foods and going to hospital.

In Malaysia, approximately 2,037,433 students (age from 13 to 15) from 2,240 schools, are currently enrolled in the secondary schools. These students were affected by the school closure as requested by the government during the Movement Control Order (MCO). For this study, student participation was solicited via online recruitment, posting of flyers around in the social media. The main criteria for the participants were selected from households of the lowest socioeconomic standing in Malaysia (family with low household income of less than RM 3,855 / 946.24 USD per month) (11). Moreover, they all voluntarily agreed to be participants for the interviews, and had full internet accesses during semi-structured interview. Consents were obtained from parents to ensure they were aware that their children were involved in this study. A total of 15 adolescents were selected as the research participants in this study. From this number, eight of them were female (53.3%) and seven of them were male students (46.6%).

The semi-structured interview is normally based on a script, where the subject matter and part of the questions have been planned before starting, but it also offers the possibility of changing or adding new questions as the interview and/or the research study moves forward, with new interviews conducted. It is the most common type of interview utilized in qualitative research on behavioral health. Semi structured interview data were gathered from 31th of March to 20th of May 2020 through online meeting apps. Participants were provided information about the study aims and protocols before beginning the interview. During the interviews, participants were asked about the impact of school closure during pandemic Covid- 19 and their behavior health experiences which shifted from general to specific matters. They were asked questions regarding their personal experiences during the MCO, and their concerns for their behavioral health during the MCO.

The recorded interviews were transcribed verbatim. Once transcribed, the interviews were imported to NVivo 12 (QSR International Pty Ltd, Doncaster, Australia). Following analysis the transcriptions, coding and themes-subthemes were discussed by the research team for their verification. Finally, participants provided feedback on the findings. The study was conducted and reported in accordance with the RATS (Relevance, Appropriateness, Transparency, and Soundness) guidelines for qualitative research (12). Table 1 summarizes four main themes highlighted by participants as key behavioral health impacts during the COVID-19 pandemic outbreak.

**Table 1:** Participant behavioral health experience during the COVID-19 pandemic outbreak.

**Essential themes**	**Thematic statement**
Changing sleep pattern and wake up time	Irregular daily activities
	Boredom
	Massive use of electronic media
Stress-related fatigue	Prolonged isolation
	Loneliness
	Repetitive daily tontines
	Inability to engage in online learning
	Piling up on homework
	Poor Internet Accessibility
Dysfunctional eating behaviors	Unhealthy snacking
	Heavy late night meal
	Improper meal time
Lack of psychical activity	Limited space
	Engagement in sedentary lifestyle
	Inadequacy of knowledge and fitness tools

## Results

The adolescents reported significant changes in sleep and waking patterns due to a lack of consistent daily routine and inadequate exposure to the outdoors. This led to them feeling listless and unmotivated in their daily lives. For instance, one of the respondents stated that: “Usually, I wake up late, feel bored because keep doing the same activities–usually sleep during the day, and at night I spend more time watching movies until late” (Respondent 1). The emotional toll of boredom led them to occupy their time with increasing use of electronic media, often until late at night, by watching movies, chatting with friends and frequently checking on the state of the pandemic. All of this disrupted their sleep patterns.

The adolescents also clarified that school closure forced them to stay at home for exhausting periods of time, restricting their ability to engage in outdoor activities and significantly cutting into their relationships, as they were socially and physically isolated from other people. This is evident when one of the respondents mentioned that:

“*feeling lonely when at home because not being able to meet friends and do physical activities together. This caused stress at home”* (Respondent 3)

As a result, they had to force themselves to live in the new normal. This situation might lead to depression as they face disappointment and loneliness. Arslan (13) justified that loneliness has a great impact on mental health, especially during the pandemics.

Furthermore, repetitive daily routines have led to adjustment issues and eventually induced stress among them. The respondents specifically mentioned that:

“*Every day I wake up, watch tv and eat, sleep… feel stress doing the same activities…. ”* (Respondent 6)

“*Before this pandemics I could create and plan various activities… but now I just stay at home and do the same activities*” (Respondent 8)

“*In fact, it is stressful to be confined for too long at home so you don't know what to do at home*” (Respondent 11)

This is because the shift from face-to-face to remote online learning at home has demanded students to adapt to ‘new' learning settings that required them to be more self-directed and independent. What is worrying, the number of home works given by their teachers has led to tiredness and triggering a sense of overwhelm and stress. To make it worse, adolescents in this low socioeconomic group reported that their house had inadequate learning facilities such as notebook or laptop and most of them are used a hand phone as a learning device. Moreover, a number of participants reported that they have a limited study area as their house was small. This has affected their motivation and comfort of studying at home.

This research also indicated that an absence of a purposeful daily routine and indistinct use of physical space, such as the separation of home and school, increased the risk of developing dysfunctional eating behaviors. This was most pronounced when adolescents felt spikes in boredom and stressful episodes at home. At these times, adolescents tended to engage in snacking or even short periods of binge eating, consuming large amounts of unnecessary calories. To support, the findings indicated that:

“*When I am bored, I will eat snacks and whatever food is in the kitchen*” (Respondent 5)

“*I always feel hungry when I stay at home so I did not notice I have gained weight because of poor eating habit* ” (Respondent 7)

In addition, participants are also felt that it was hard to control their eating behaviors, especially night time habits. On the opposite end of the spectrum, half of the adolescents stated that they had inadequate sources of food and nutrition because of constraints in family finances and more mouths to feed.

“*When at home, the meal time is unpredictable and I only eat what is available… my father has to be frugal to spend because he does not have enough money to buy nutritious food*.” (Respondent 7)

“*I have many siblings, so we have to share food and sometimes I only eat once a day because there is not enough food*” (Respondent 13)

These individuals experienced difficulty with proper meal time routines because they needed to limit the number of meals per day. This was a contributing factor toward irregular eating behaviors.

Adolescents from low-income households explained that they were vulnerable toward the negative sides of staying at home because they did not have standard, designated space for themselves. They felt it was difficult to engage in physical activity. One of the respondents specifically mentioned that: “…the space of my house is small and has to be shared with other siblings. So, we are restricted to do physical activities at home” (Respondent 14). They claimed that their lifestyle was mostly inactive, predominated by sedentary activities such as surfing social media, irregular napping, waking late, and staying at home for longer time than they used to. They were more inclined to channel their boredom by eating unhealthily and thus has contributed to weight gain. Since they were still learning to adapt to the new normal, they found it difficult to start physical activities at home.

## Discussion

The study shows the impact of school closure and prolonged isolation on the behavioral health among adolescents at home. Results indicated that there were four main aspects of behavioral health faced by adolescents during the COVID-19 pandemic, namely: changing sleep patterns, stress-related fatigue, dysfunctional eating behaviors and lack of physical activity. It shows that adolescents require support in managing and balancing these four aspects of behavioral health during the pandemics. Thus, this highlights the need for educating parents and adolescents on behavioral health to ensure stable and positive coping skills among adolescents. In addition, adequate knowledge and skills on behavioral health are needed to better prepare the adolescents to face the challenges in a pandemic. In doing so, awareness and deeper understanding on how to manage this pressing situation is highly needed. Consistent, high-quality sleeping habits safeguard physical and mental health are important to reduce the risks of stress (14, 15).

Further, adolescents require support in managing their emotions. Other studies have found that such lifestyle and social life changes impact adolescent emotional growth and may lead to depression (16–18). Emotional management skills are therefore important for adolescents to develop. While staying at home, they need to identify and plan focused, beneficial activites that can enhance their positive emotions. Education could also help individuals creatively optimize limited house space to engage in physical activities that promote incidental and intentional physical exercise, and reduce the risks of obesity (19, 20). School administrations should also support health education via practical, creative and targeted videos to help increase awareness about healthy eating, nutrition, sleep and other aspects of behavioral health (21). Mass media and social media may also play significant roles in this situation by disseminating consistent updates on assistance that the stakeholders such as government agencies, educational institutions, and medical institutions provide to meet daily necessities, such as food, for those most severely impacted by the MCO.

## Conclusion

This study put forward the issues of behavioral health among adolescents as a result of urgent and sudden school closures. There is a need to improve the health communication that exists among adolescents to increase their awareness on knowledge and practice of proper behavioral health during the pandemic. As of March, although COVID-19 vaccinations are underway around the world, the threat from new variants as well as the general fear of a surge, still means that the MCO and similar restrictions may persist for much longer. It is important for schools and parents to foster awareness and the importance of behavioral health management at home.

Furthermore, the uncertainty of the situation also requires consistent messaging from mass media, in order to disseminate a wide range of public health messages to increase knowledge and strategy of how to nourish behavioral well-being at home, as well as to prevent COVID-19 infection. The findings of this study may serve as important reference for policy makers to help foster awareness and knowledge on how to manage their behavioral health effectively at home. In conclusion, with the improvements in communication strategy upgraded in disseminating on behavioral health helps to give space for adolescents to gain access on information to increase their behavioral well-being. Further research in understanding this pressing situation. Since the current study only included students from the lowest social economic status, it is recommended to include students from other social economic status. This will provide a deeper understanding of unplanned school closure impacts on them.

## Data Availability Statement

The original contributions presented in the study are included in the article/supplementary material, further inquiries can be directed to the corresponding author/s.

## Ethics Statement

The studies involving human participants were reviewed and approved by Jawatankuasa Penyelidikan 3M Faculty of Education. Written informed consent to participate in this study was provided by the participants' legal guardian/next of kin.

## Author Contributions

MA: conception and design of the work. MA and KJ: acquisition, analysis of data, interpretation of data, drafting the work, and revising the final approval of the work.

## Conflict of Interest

The authors declare that the research was conducted in the absence of any commercial or financial relationships that could be construed as a potential conflict of interest.
